# Improving patient flow through an eye clinic

**Published:** 2012

**Authors:** Jonathan Pons

**Affiliations:** Ophthalmologist and Programme Director, Good Shepherd Hospital Eye Care Project, PO Box 218, Siteki, Swaziland. Email: jono@goodshepherdhosp.org

**Figure:**
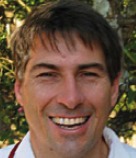
Jonathan Pons

Improving the flow of patients through our eye programme is about making their journey easier while making the best use of our own time and resources in the eye clinic. It involves elminating unnecessary steps and processes, giving us more time to focus on our patients and on providing a good – and friendly -service. Eye care administrators and managers benefit too: better patient flow reduces waste and makes more efficient use of theatre time and human resources, which in turn reduces costs, attracts more patients, and improves cost recovery.

Thinking about what our patients value can help us to optimise patient flow. Generally speaking, patients value everything that provides them with a good outcome: appropriate referral, a correct diagnosis, the right information and advice, the right treatment, and appropriate follow-up and aftercare. They do not value things that seem unnecessary to them, for example: waiting longer than seems reasonable, having to provide the same information more than once, or travelling to the hospital more than once when two visits can be safely combined.

It is therefore very helpful to look at our eye service as a whole from time to time, particularly if we have received negative feedback from our patients. We must examine everything we do: from the moment of first contact with our patients to the time they are finally discharged after a successful follow-up examination.

The good news isthat, by thinking about our patients and how to provide them with a good experience in our clinic, we will be able to make changes that benefit the the clinic as well. See Table 1 for some examples.

**Figure:**
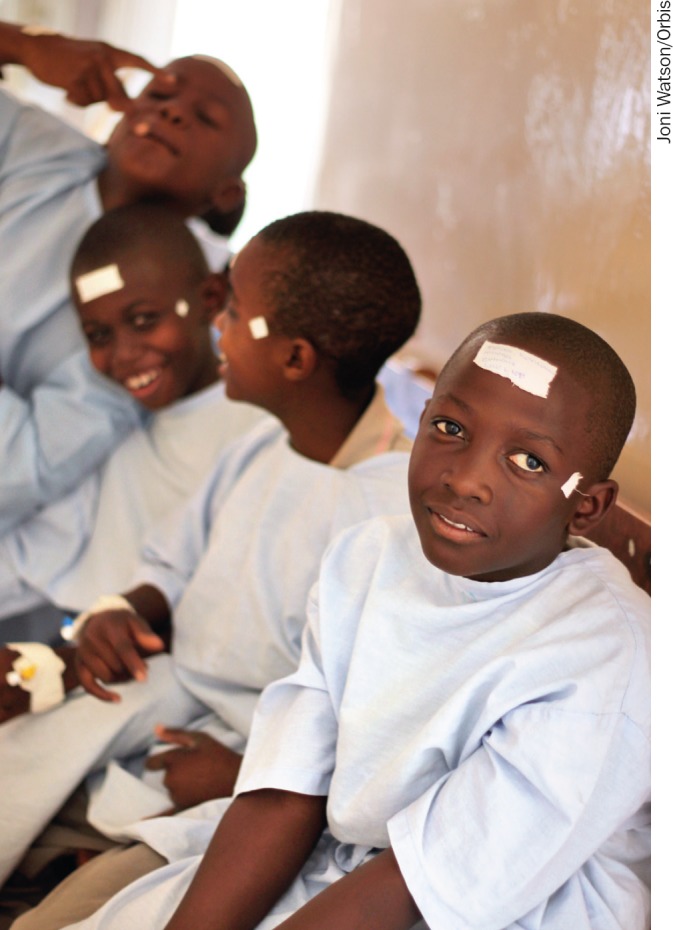
Preparing groups of cataract patients for surgery means that scarce resources such as theatre time can be used more efficiently. ZAMBIA

## The patient journey

It helps to consider the patient's visit to the eye clinic as a journey. Here are some examples of the different ‘stations’ along a patient journey through an eye clinic:

- Registration
- Retrieval of medical records
- Visual acuity testing
- Slit lamp examination Consultation
- Treatment
- Fee collection

If we want to consider how a patient is referred to our clinic, particularly if our clinic forms part of a VISION 2020 district programme or a government district health care system, we can include steps such as Outreach’, ‘primary health care referral’ and so on in the list above.

## Understanding existing patient flow

Many patients will travel through our eye clinics and it is our responsibility see that patient flow is well managed. Before making any improvements, start by assessing (or auditing) the existing patient flow in the eye clinic. This can be done by one person, but it is often better to invite representatives from both clinical and support staff to help. Everyone's input is valuable.

Regular evaluation of patient flow will allow us to identify problems and make helpful changes. The suggestions that follow overleaf should help you to start thinking about patient flow and identify areas for improvement.

The focus should be on what patients value: does the way the clinic function help us to give patients the best service we can?

**Table 1.** How Improving patient flow can benefit patients and the eye programme: a few examples

What patients want	What the eye unit wants	How improving patient flow could meet the needs of patients and the eye hospital
Less waiting time	Efficient use of staff time	If some staff are waiting for patients, find areas where patients are waiting for staff and move the staff to that part of the process.
Lower prices for eye care	Reduced waste	Eliminate any unnecessary procedures or diagnostic tests, provided they do not affect the quality of clinical care
Good quality care	Sharing of scarce resources, e.g. slit lamps or theatre time	Prepare patients for examination or theatre in a separate area so that the time spent at the slit lamp or in theatre is kept to a minimum.
Lower travel costs, less time away from home	Reduce patients who do not attend for operations or who do not come for pre-operative examinations	Where possible, do pre-operative examinations on the same day as the operation.
Respect and care	Co-operative patients, enough time to provide proper care, a good reputation	Provide information at the start of the patient's journey about what is likely to happen, how long it might take, and how much it is likely to cost. This puts patients at ease, so staff can focus on what is important.

- List the different ‘stations’ on a typical patient's journey through your clinic. How long do they have to wait before moving through each station? You could assign a staff member or volunteer to visit waiting areas and monitor the waiting times. What do patients think? You could conduct exit interviews with patients or consider assigning a staff member or volunteer to do patient shadowing (see page 23).
- Look at the patients’ physical journey through the clinic. On a detailed plan of the clinic, trace the paths they have to walk between each of the stations. Are there any unnecessary back-and-forth movements? Do patients know where to go? Do staff often have to stop what they are doing and help direct patients?
- Trace the paths different staff members have to take as they carry out their various daily tasks. Include support staff as well, such as administrators, porters, stock room staff, etc. Ask staff: is there anything that could be changed to make their work easier?
- Look at the use of equipment. Is there enough equipment? Is unused equipment taking up valuable space in the passageways or consulting rooms?
- What are the times and days of the week, month, or year when the clinic is busiest?
- Look at the procedures for stores and purchasing, and at how you keep records and identify patients (see ‘Further reading’ on page 35). Are patients required to provide the same information more than once?

## Knowing what to change

There are various approaches to analysing patient flow, with names like ‘process mapping’ and ‘value stream mapping’ (see ‘Further reading’ on page 35).

Finding and eliminating bottlenecks is another approach and is relatively straightforward. The aim is to reduce waiting times and make better use of equipment and the time of clinicians.

Bottlenecks are usually easy to identify: they are the areas with the longest queues! For example, one often sees long queues in front of the visual acuity testing station, whereas, in another part of the clinic, the screening station is waiting for patients. In this instance, the visual acuity testing station is the bottleneck – it is the part of the clinic where patients are getting stuck.

**Figure:**
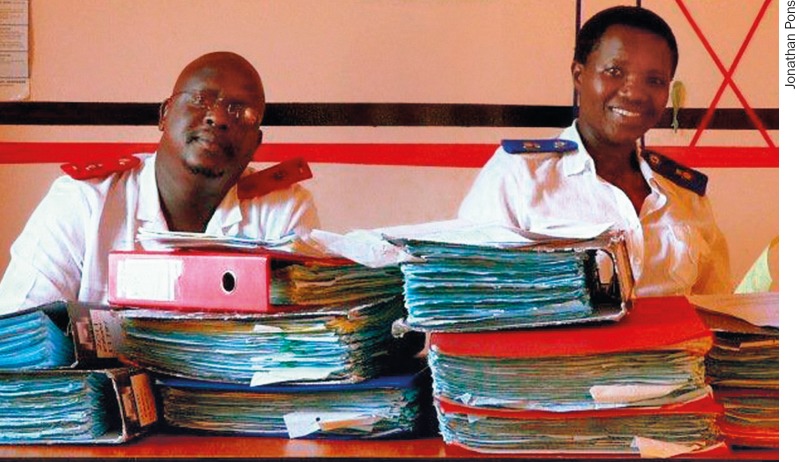
Good record keeping reduces delays and improves patient flow. SWAZILAND

Using an additional person at the visual acuity testing stage would speed up the flow of patients through this area and provide a steady stream of patients at the screening station. Patients will therefore have a quicker journey, and eye care workers' time will be used more efficiently.

It is worth noting that this isa process of ongoing improvement: once one bottleneck has been dealt with, it will very soon become clear if another part of the clinic has become congested and will require attention.

## How to make changes

Once we better understand patient flow in our eye clinic, and where the delays and inefficiencies are, the next step is to talk to clinical and support staff about how improvements can be made.

**CASE STUDY: KILIMANJARO CHRISTIAN MEDICAL CENTRE (KCMC), TANZANIA: Doing a baseline assessment**

The team responsible for leading the changes at KCMC used a baseline assessment form produced by Lions Aravind Institute for Community Ophthalmology to help them understand the eye department's resources and problems. Problem areas included:

- **Inconvenience for patients:** the system for a patient to be registered for outpatients or admission was lengthy and complicated.
- **Personnel used inefficiently:** many nurses spent more time on clerical duties than on nursing, and doctors were often responsible for mundane management tasks.
- **Inefficient procedures:** there were no standard clinical protocols for common problems like cataract.
- **Monitoring:** basic annual patient service statistics were collected, but these were not discussed with staff.
- **Stores and purchasing:** there was no system for making stores reports and none were made; there was no system for efficient purchasing.

It is important to create an atmosphere of teamwork and collaboration, and to encourage everyone to contribute their ideas. Janitors or stock control clerks, for example, may offer valuable insights into everyday processes that can be streamlined.

Giving staff an opportunity to contribute has the added advantage of making staff members feel like part of a team; agreeing on a shared goal also makes it easier for people to work together.

## Practical suggestions

Becoming better organised allows us to make better use of available clinic space and infrastructure and to make better use of staff time.

This can often avoid or delay the need for an expensive expansion programme!

Here are some practical ideas for improving patient flow.

### Better systems

- Standardise procedures in the clinic. This will allow more patients to be seen in a day and make it easier to keep quality consistent.
- Use tags or stickers on charts to make them easy to identify.
- Make use of helpful technology where appropriate. For example, use computers for indexing records or use devices that will speed up intraocular pressure readings.
- Some days are busier than others (e.g., Mondays are usually busier because of weekend emergencies). Part of a solution to an overcrowded clinic may involve moving clinic activities to different days to allow a better spread of patients throughout the week.
- To reduce unnecessary back and forth movement of patients because of multiple payments to cashiers, try to offer ‘package’ prices that cover the cost of multiple services. Or set up a system that allows patients to pay when they leave for all the services they have used.
- Good internal communication systems (intercoms, oran intranet) between the various departments will make it easier to share information about patients and will also save time (see panel on right for an example from Madagascar).

### Better use of space

- Arrange the different stations in the patient journey (registration, records retrieval, visual acuity testing, etc.) in a logical sequence so that patients can easily move from one to the next.
- Put related services nearby. Sometimes, something as simple as moving an optometrist into the clinic can make a big difference to patients!
- Try to avoid any back and forth movements, where patients have to cross paths with others, as this can create confusion. When a room has just one door, patients who are leaving may have to squeeze past patients who are queuing to get in. Use two doors or, if need be, open up a new doorway in an existing wall.
- Clearly signpost each station in the clinic so patients know whether they are at the right place. Paint doors different colours or number them in a large font. Drawings are particularly helpful for patients who cannot read.
- Use colour-coded lines on the floor to help direct patients to different stops along their journey.
- Locate cashiers and drug dispensaries at the outlet of the clinic in order to avoid unnecessary back and forth movements of patients; this reduces congestion.
- Have staff available to help patients who cannot find their way.
- Sometimes, using two rooms can reduce waiting times. For example, while an ophthalmologist is busy with a consultation in one room, a nurse or nurse assistant could get a patient ready at a slit lamp in the room next door.

### Better use of staff

- Make good use of mid-level ophthalmic personnel, nurses, and nursing assistants. They are usually highly trained and can perform many tasks that will free up the time of ophthalmologists so they can focus on what only they can do.
- Make more staff available during busy times, and stagger lunch breaks so that work flow is continuous. This will reduce patients’ waiting times.
- Encouraging a culture of teamwork will help to improve patients’ experience at the clinic. Treating staff fairly and with respect will reduce the likelihood of interpersonal problems.
  **AN INTEGRATED INFORMATION AND COMMUNICATION SYSTEM**

**Figure:**
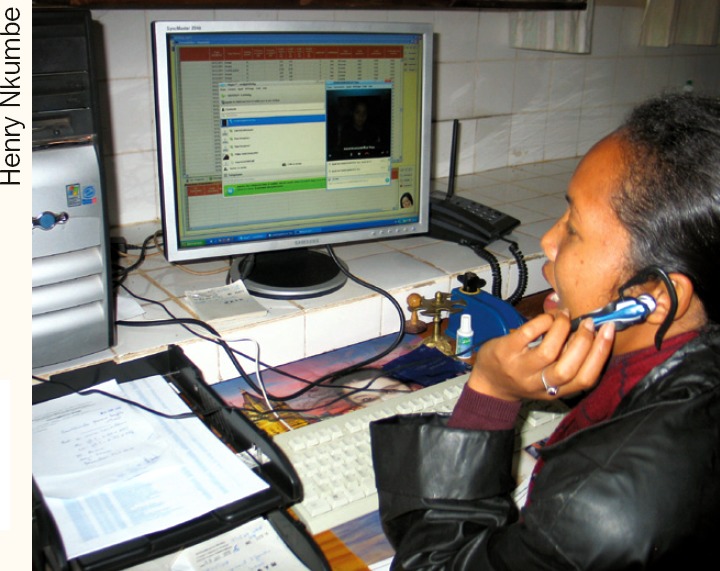

  SALFA Eye Clinic in Fianarantsoa, Madagascar, has introduced a fully integrated electronic medical records system recently. All major stations (reception, pharmacy, cashier, ophthalmologists, manager, stores, etc.) have a Computer terminal, headset and webcam. This allows for quicker and cheapercommunication: staff can talk to and see each other free of charge using Skype, a free software package that requires internet access in order to work **(www.skype.com)**. The records system also gives the clinic manager, Somoela Rajaona, all the information he needs to manage patient flow. For example, he can see how many patients are waiting to be seen by the doctor, the stock situation, and so on.
- Problems in how staff are managed can lead to poor team morale. Staff who are happy, and feel respected by their colleagues or managers, find it easier to be kind and friendly to patients and to contribute to clinic improvements.

### Other problems that affect patient flow

There are some problems that affect the entire patient journey.

- Inefficient recordkeeping can cause many delays. A records retrieval rate of less than 90% should not be tolerated in an eye clinic! Periodic review of all forms and stationery is useful; check that patients do not have to provide the same information more than once, unless absolutely necessary.
- Patients and clinic staff who do not understand each other's language is another common problem. Take steps to ensure that essential patient education materials are available in a local language, particularly instructions for medication. Where possible, ensure there are sufficient interpreters available. Ask for help from local churches or community organisations.

## An ongoing journey

A patient's journey does not end when she or he leaves our clinic. Good referral to other services, such as low vision or rehabilitation clinics, must form part of the service you offer.

The spacing of follow-up visits should also reflect the patient's situation and balance the need for good clinical care with the ability of patients to travel to the clinic. Clearly indicate the date of any follow-up visits on the patient's records, and send reminder messages by cellphone (mobile phone) if possible.

Optimising patient flow is a journey of ongoing improvement. We hope that this article has helped you take the first steps.

**CASE STUDY: KILIMANJARO CHRISTIAN MEDICAL CENTRE (KCMC), TANZANIA: Improvements on the ward and in theatre**

Once the new community outreach programme started bringing in large numbers of patients, especially late in the day, the need to make ward and theatre procedures more efficient became critical. The team decided that it would be more efficient if the counsellor (a trained nurse) working in the outreach programme recorded vital signs, completed consent forms, and educated the patients right there in the field. As a result, the ward nurses had less to do at the time of admission. New forms, designed by an external nurse consultant working with the eye department nurses, also saved time. In the operating theatre (OT), improving efficiency was partly a matter of clearing unnecessary equipment and supplies from the OT so that an extra operating table could be installed. It also required many discussions with the doctors as to how the OT should be run and the importance of starting on time.

Under the leadership of the nursing co-ordinator, and motivated by positive feedback and praise from the head of the ophthalmology deparment, more nurses began to take pride in their accomplishments; this was a modest but important step forward in achieving better attitudes and motivation.

